# Effects of carbon and nitrogen nutrition characteristics in vegetative parts on grain yield in maize

**DOI:** 10.3389/fpls.2025.1751019

**Published:** 2026-01-21

**Authors:** Lin Shi, Linzheng Liao, Guowei Chen, Yuping Wang, Yanzhi Tan, Yahan Zhang, Fang Yu, Meng Lin, Qiang Li, Yun Ren, Xuewei Yin

**Affiliations:** 1 Chongqing Key Laboratory for Germplasm Innovation of Special Aromatic Spice Plants, College of Smart Agriculture/Institute of Special Plants, Chongqing University of Arts and Sciences, Chongqing, China; 2 Special Crop Research Institute, Chongqing Academy of Agricultural Sciences, Chongqing, China

**Keywords:** accumulation and distribution, carbon nutrition, maize, nitrogen nutrition, yield

## Abstract

We investigated the effects of nitrogen application on carbon and nitrogen nutrition characteristics of maize nutritional organs and grain yield, and clarified the relationship between carbon and nitrogen nutrition characteristics of maize nutritional organs and grain yield. The field trials were conducted from 2019 to 2020. The nitrogen-efficient Zhenghong 311 (ZH 311) and nitrogen-inefficient Xianyu 508 (XY 508) varieties were used as experimental materials. We used four nitrogen fertilizer rates (0, 120, 240, and 360 kg ha^−1^), labeled as N1-N4 (with N1 being the 0 kg N ha^−1^ control). The results indicated that synergistic regulation of carbon-nitrogen metabolism plays a crucial role in yield formation, and the rationale lies in the differential responses of maize varieties to nitrogen levels, with key findings showing that ZH 311 achieves high yield under moderate nitrogen (N) conditions while XY 508 depends on high N supply. At maturity, ZH 311 exhibited a higher nitrogen content in culm sheaths, leaves, and ears than XY 508, whereas root nitrogen content was lower than that of XY 508. However, carbon content differences among organs at maturity were not significant between varieties, resulting in lower C/N ratios in ZH 311 organs at maturity than those in XY 508. The C/N ratios in maize nutritional organs were negatively correlated with yield, indicating that maintaining a low nutritional organ C/N ratio is a key mechanism for nitrogen-efficient varieties to achieve higher yields than nitrogen-inefficient varieties. Nitrogen application significantly increased carbon and nitrogen accumulation in the maize nutritional organs. However, the increase in all organs was greater in XY 508 than in ZH 311, indicating that additional nitrogen fertilizer is more beneficial for enhancing carbon and nitrogen accumulation in the nutritional organs of nitrogen-inefficient varieties. Moreover, nitrogen-inefficient varieties require higher nitrogen application rates to maintain carbon and nitrogen accumulation. However, nitrogen-efficient varieties exhibited substantially higher carbon and nitrogen accumulation in the organs than the nitrogen-inefficient varieties at identical nitrogen levels. Correlation analysis revealed that carbon and nitrogen accumulation in the nutritional organs were positively correlated with grain yield at maize maturity. Leaf nitrogen accumulation (*R*² = 0.8641**) showed the strongest correlation with grain yield, whereas stem sheath carbon accumulation (*R*² = 0.8257**) exhibited the highest correlation with grain yield.

## 1 Introduction

Maize (*Zea mays* L.) is the world’s foremost staple crop, with annual global production exceeding 10×10^8^ tons, accounting for over 40% of the world’s total grain output. High and stable maize yields are crucial to safeguard global food security (Qi et al., 2023; Zhou, 2023; Zhou et al., 2024). China cultivates maize across 43.32 million hectares, yielding 27300 tons, representing over 23% of the global maize production and ranking second worldwide (Zhang et al., 2023). Nitrogen fertilization is fundamental to achieving high, stable maize yields. Traditional fertilization practices in China typically yield nitrogen utilization rates of less than 30%. Optimizing fertilization methods (nitrogen application rates and basal-to-top dressing ratios) can enhance nitrogen use efficiency (Zhang, 2022), although this improvement remains limited. This efficiency still lags significantly behind the 50–60% rate achieved in developed nations. Further exploration of the biological characteristics and genetic improvement of maize is essential to enhance nitrogen efficiency substantially. Clarifying the physiological mechanisms underlying nitrogen efficiency variations forms a foundation for improving the efficiency of maize nitrogen use.

Carbon and nitrogen metabolism, the two primary metabolic pathways in plants, are jointly modulated by genetic and environmental factors, with their coordination serving as a core determinant of yield formation (Lei et al., 2024). Carbon metabolism supplies carbon sources and energy to nitrogen metabolism, which provides enzymes and photosynthetic pigments for carbon metabolism. Both pathways share the requirements for reducing power, ATP, and carbon skeletons, thereby exhibiting mutual influence and interdependence. Yu et al. (2014) noted that rapeseed varieties exhibiting robust carbon and nitrogen metabolism demonstrated higher nitrogen use efficiency. Furthermore, varieties with elevated nitrogen use efficiency accumulated greater carbon and nitrogen reserves and yielded higher outputs, indicating a close correlation between carbon-nitrogen metabolism and rapeseed yield. Nitrogen-efficient maize varieties balance carbon and nitrogen metabolism better than nitrogen-inefficient varieties, enhancing their adaptability to low nitrogen stress. Maize yield showed a significant positive correlation with both carbon and nitrogen turnover rates; higher turnover rates increased yield and nitrogen fertilizer utilization efficiency (Wu et al., 2019). Increased nitrogen fertilization improves wheat carbon–nitrogen metabolism and enhances carbon and nitrogen accumulation and transport. This increases the number of effective spikes, grains per spike, and thousand-grain weight, thereby significantly boosting the yield (Li, 2023a). In summary, crop grain yield is closely linked to the accumulation, distribution, and balance of carbon and nitrogen nutrients.

Previous studies on maize carbon and nitrogen nutrition characteristics have predominantly focused on differences in enzyme activity and marker compound (soluble sugars and amino acids) content, with most investigations conducted using pot-grown seedlings. However, the mechanisms underlying variations in carbon and nitrogen content, accumulation, allocation, and balance within nutritional organs across maize varieties with differing nitrogen efficiencies and their impact on yield remain unclear (Lv et al., 2023; Jiao et al., 2020). Therefore, we used maize varieties with differing nitrogen efficiencies (the nitrogen-efficient varieties Zhenghong 311 and ZH 311, and the nitrogen-inefficient varieties Xianyu 508 and XY 508) as experimental materials. Under field conditions, four nitrogen fertilizer levels (0, 120, 240, and 360 kg ha^−1^) were used to investigate variations in carbon and nitrogen content, accumulation, allocation, and balance across organs of these maize varieties, along with their relationship to grain yield. This study aims to elucidate differences in carbon (C) and nitrogen (N) accumulation, allocation, and balance in the nutritional organs of maize varieties with varying nitrogen use efficiency, and to establish their relationship with yield. It provides a physiological basis for genetic improvement of nitrogen-efficient maize and precision nutrient management in the purple soil regions of Southwest China, thereby facilitating the selection of nitrogen-efficient varieties and the formulation of site-specific fertilization strategies for this area.

## 2 Materials and methods

### 2.1 Experimental materials

The trial used the nitrogen-efficient variety Zhenghong 311 (ZH 311) and the nitrogen-inefficient variety Xianyu 508 (XY 508) as test materials. Both varieties are primary maize cultivars in the southwestern region and exhibit a largely consistent growth period of approximately 120 d.

### 2.2 Overview of the experimental site

**Figure 1:**
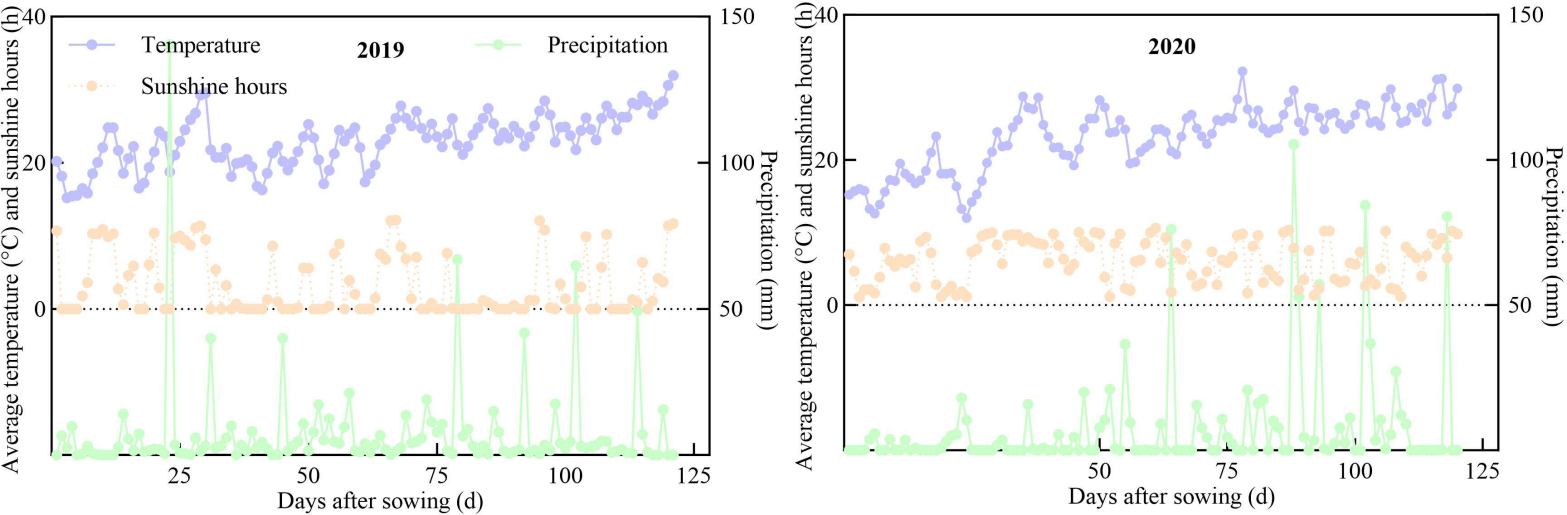
Meteorological factors during maize growth.

The trial was conducted in Yongchuan, Chongqing (29°21’N, 105°54’E) during 2019–2020. This region exhibits a subtropical monsoon climate with meteorological factors during maize growth periods, as shown in (**Figure 1**). The experimental soil was purple soil. The 0–30 cm soil nutrient levels in 2019 (2020) were as follows: pH 7.92 (7.63), total nitrogen 1.63 g kg^−1^ (1.55 g kg^−1^), total phosphorus 0.62 g kg^−1^ (0.49 g kg^−1^), total potassium 11.55 g kg^−1^ (9.74 g kg^−1^), available nitrogen 48.72 mg kg^−1^ (39.77 mg kg^−1^), available phosphorus 2.68 mg kg^−1^ (2.37 mg kg^−1^), available potassium 145.21 mg kg^−1^ (131.19 mg kg^−1^), and organic matter 16.14 g kg^−1^ (13.97 g kg^−1^).

### 2.3 Experimental design

The trial used a two-factor randomized block design. Factor one was the maize variety: the nitrogen-efficient ZH 311 and the nitrogen-inefficient XY 508. Factor two was nitrogen fertilizer rate: N1:0 kg ha^−1^, N2:120 kg ha^−1^, N3:240 kg ha^−1^, N4:360 kg ha^−1^. Each treatment was replicated thrice, comprising 24 plots, each measuring 5×8 m (40 m²). Maize was planted in wide-narrow rows (1.4 m + 0.6 m) at a density of 52,500 plants ha^−1^; uniform seeds were manually sown at 3–5 cm depth post soil preparation, with thinning and gap-filling at the 3-leaf stage to ensure target density. Nitrogen fertilizer was applied according to the experimental treatments, with equal amounts applied as basal fertilizer before sowing and as a top-dressing at the large-flute stage. Phosphorus and potassium fertilizers were applied as basal dressings in a single application: superphosphate at 600 kg ha^−1^ and potassium chloride at 150 kg ha^−1^, all basal fertilizers were rototilled into 0–20 cm soil 1 day before sowing. Topdressing N was applied in equal amounts at the large-flute stage; it was furrow-applied 10 cm from rows and covered immediately to reduce volatilization. Local high-yield cultivation practices were followed for pest, disease, and weed control. Manual weeding was conducted twice (seedling and before sowing stages). Integrated pest and disease management (IPM) was implemented, including bacillus thuringiensis for maize borers/armyworms at seedling stage (supplemented with carbaryl when pest populations exceed economic thresholds) and propiconazole foliar spray for early northern maize leaf blight. Drip irrigation was initiated when 0–20 cm soil moisture < 60% field capacity, especially at tasseling, silking and grain filling stages.

### 2.4 Measurement and methods of indicators

At maize maturity, five representative plants were selected from the middle row of each plot based on average plant height. Samples were separated into roots, stem sheaths, leaves, and ears. All samples were blanched at 105°C for 30 minutes, dried at 80°C to constant weight, then weighed, pulverized through a 60-mesh sieve, and set aside for later use. Total carbon (C) and nitrogen (N) contents were determined using an elemental analyzer (Vario Max CN, Elemental, Germany).

At maturity, 20 plants were selected from each plot for grain measurements to determine the number of rows per ear, grains per row, grains per ear, and thousand-grain weight. All plants within a 10 m² area, excluding those in the edge rows, were harvested for yield assessment. All grains were naturally sun-dried, and the yield and thouand-grain weight were measured. The moisture content was converted to 14.0%, moisture conversion standard (GB/T 15671-2019).

### 2.5 Data statistics and analysis

Data were organized using Excel 2019 and analyzed using the SPSS software package (version 20.0, IBM Corp., Armonk, NY, USA), using the least significant difference (LSD) test to determine whether significant differences existed between varieties, nitrogen levels, and their interactions, with the significance level standardized at *p* < 0.05. Graphical representations were generated using GraphPad Prism software (version 5.0, GraphPad Software, San Diego, CA, USA).

## 3 Results

### 3.1 Differences in dry matter accumulation among organs in maize varieties with varied nitrogen use efficiency

Dry matter accumulation in maize organs at maturity was the highest in the ear and lowest in the root system (**Table 1**). Both variety and nitrogen fertilizer significantly influenced the dry matter accumulation in maize roots, stem sheaths, leaves, ears, and total dry matter. ZH 311 exhibited significantly higher (*p* < 0.01) dry matter accumulation in the roots, stem sheaths, leaves, ears, and total dry matter than XY 508 across both years. In 2019, these values were 9.46%, 42.23%, 36.16%, 26.73%, and 28.88% higher, respectively, while in 2020, the increases were 15.19%, 22.43%, 53.63%, 16.79%, and 21.54%, respectively. This indicates that ZH 311 has a clear advantage over XY 508 in terms of dry matter accumulation across all plant parts. Nitrogen application significantly increased (*p* < 0.01) dry matter accumulation in the roots, stem sheaths, leaves, ears, as well as total dry matter for both varieties. For ZH 311, dry matter accumulation in roots, stem sheaths, leaves, and ears, as well as total dry matter, initially increased and then decreased with increasing nitrogen application across both years, peaking in the N3 treatment. Conversely, XY 508 showed consistent increases in response to nitrogen application across both years, reaching maximum levels in the N4 treatment group. Consequently, ZH 311 exhibited greater advantages than XY 508 in terms of roots, stem sheaths, leaves, ears, and total dry matter accumulation in ZH 311 over XY 508, which initially increased and then decreased with increasing nitrogen application rates, peaking in the N2 treatment. This indicates that the nitrogen-efficient variety ZH 311 possesses a greater dry matter production advantage than the nitrogen-inefficient variety XY 508 at medium-to-low nitrogen levels.

**Table 1:** Dry matter accumulation in different organs of maize (t ha^-1^).

Cultivars	Nitrogen rate	Root		Stem+sheath		Leaf		Ear		Dry matter accumulation	
		2019	2020	2019	2020	2019	2020	2019	2020	2019	2020
ZH 311	N1	1.31bcd	1.09 cd	2.50 d	2.69 d	1.39 bc	1.57 b	6.91 e	6.46 d	12.11 e	11.82 e
	N2	1.51 ab	1.30 b	3.52 b	2.88 cd	1.85 a	1.80 a	9.27 b	7.97 c	16.14 b	13.95 c
	N3	1.65 a	1.53 a	4.03 a	3.41 a	2.00 a	1.92 a	10.29 a	9.17 a	17.97 a	16.03 a
	N4	1.50 ab	1.36 b	3.78 ab	3.14 b	1.84 a	1.85 a	9.72 b	8.72 ab	16.84 b	15.07 b
	Mean	1.49 A	1.32 A	3.46 A	3.03 A	1.77 A	1.79 A	9.05 A	8.08 A	15.77 A	14.22 A
XY 508	N1	1.15 d	1.01 d	1.86 f	2.01 e	1.05 d	0.90 e	5.52 f	5.11 e	9.59 g	9.03 g
	N2	1.28 cd	1.11 cd	2.20 e	2.20 e	1.26 c	1.14 d	6.86 e	6.41 d	11.21 f	10.86 f
	N3	1.46abc	1.25 bc	2.74 cd	2.77 cd	1.35 c	1.26 cd	7.73 d	7.94 c	13.28 d	13.23 d
	N4	1.56 a	1.21 bc	2.93 c	2.92 bc	1.53 b	1.34 c	8.45 c	8.22 bc	14.46 c	13.69 cd
	Mean	1.36 B	1.14 B	2.43 B	2.47 B	1.30 B	1.16 B	7.14	6.92 B	12.14 B	11.70 B
F value	Years (Y)	42.69**		20.29**		5.54*		49.78**		58.18**	
	Cultivar (C)	25.36**		349.26**		486.37**		334.01**		559.11**	
	Nitrogen (N)	23.11**		117.73**		60.13**		245.75**		263.96**	
	Y × C	0.58ns		31.21**		9.78**		19.66**		18.42**	
	Y × N	0.32ns		7.94**		1.17ns		1.46ns		2.36ns	
	C × N	2.08ns		7.22**		5.01**		9.20**		14.43**	
	Y × C × N	1.31ns		4.19*		2.06ns		2.56ns		3.73*	

*presents significant difference at (*p* <0.05), **presents significant difference at (*p* <0.01), NS, nonsignificant.

Lowercase letters (a, b, c, d) following numerical values (e.g., 1.31bcd, 1.09cd): These letters represent the results of Duncan’s multiple range test (P < 0.05) for comparing the means of dry matter accumulation under different nitrogen application rates (N1–N4) within the same organ and same year. Values marked with the same lowercase letter are not significantly different, while different letters indicate statistically significant differences.

Gray background of the “Mean” row: This formatting is used to visually distinguish the average dry matter accumulation (across all nitrogen rates) from the individual treatment values, helping readers quickly identify and compare the overall average levels of each organ.

Uppercase letters (A, B) in the “Mean” row (e.g., 1.49 A, 1.63 A): These uppercase letters also reflect Duncan’s multiple range test (P < 0.05), but for comparing mean values between the two experimental years (2019 vs. 2020). The same uppercase letter indicates no significant difference between years, while different letters denote a significant difference.

### 3.2 Differences in carbon and nitrogen content among organs of maize varieties with varied nitrogen use efficiency

**Figure 2:**
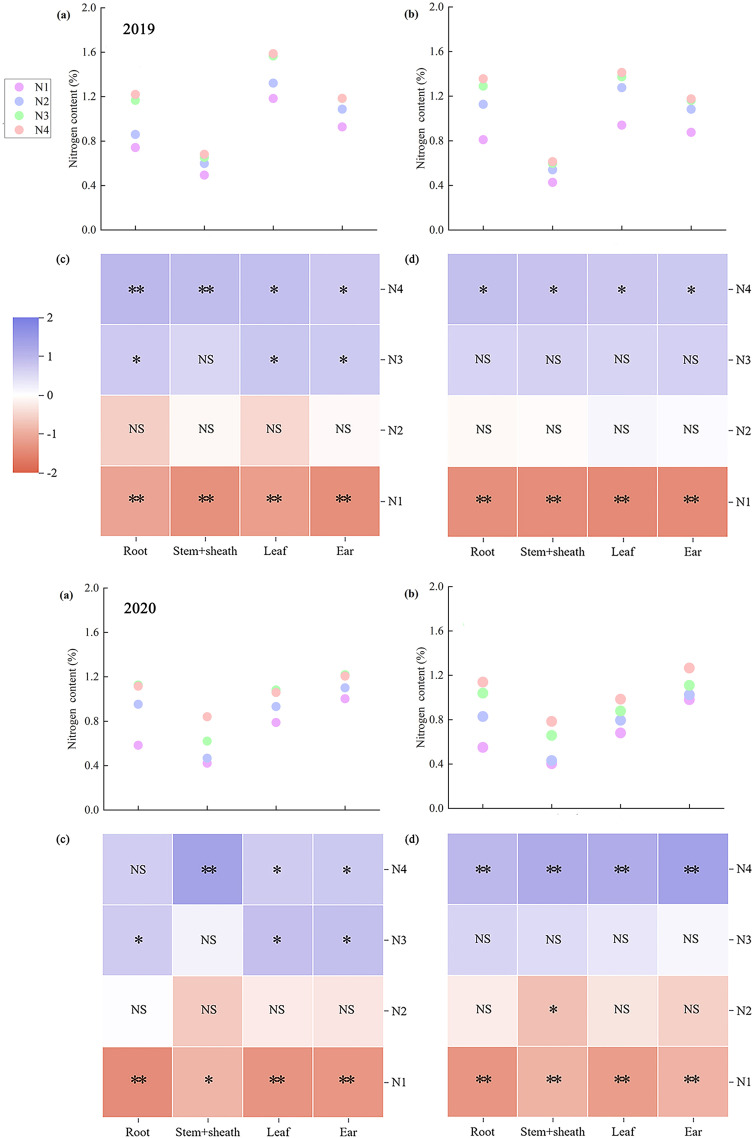
Nitrogen content in different organs of maize under different treatments. An asterisk indicates a significant difference between the sample homogenization *t*-test and the mean. **(a)** and **(c)**: ZH 311, **(b)** and **(d)**: XY 508, *p < 0.05, **p < 0.01, and NS, nonsignificant, the same below (**Figures 3**–**5**).

The nitrogen content varied significantly across maize organs at maturity. Across both years, root nitrogen content showed the greatest variation, whereas the stem sheath nitrogen content showed the smallest variation. Leaf nitrogen content peaked in 2019, whereas ear nitrogen content was the highest in 2020. The stem sheath nitrogen content was the lowest across both years (**Figure 2**). Across all nitrogen application treatments, ZH 311 exhibited higher nitrogen content in the roots (except in 2019), stem sheaths, leaves, and ears than XY 508. In 2019, these differences amounted to 13.00%, 11.40%, 13.08%, and 1.99%, respectively, whereas in 2020, they were 6.23%, 3.28%, 15.67%, and 3.39% higher, respectively. This indicates that ZH 311 maintains a higher nitrogen content in all organs at maturity than XY 508 does, thereby delaying organ senescence. Nitrogen application elevated the nitrogen content in all maize organs at maturity, with pronounced varietal differences. Nitrogen treatment induces greater variability in ZH 311. Compared to the N1 control, all nitrogen treatments increased nitrogen content in roots, stem sheaths, leaves, and ears by 45.77%, 30.35%, 26.06%, and 24.32%, respectively, in ZH 311 exhibited increased nitrogen content in roots, stem sheaths, leaves, and ears by 45.77%, 30.35%, 26.06%, and 24.32%, respectively, in 2019, and by 82.79%, 52.38%, 29.96%, and 17.22%, respectively, in 2020; For XY 508, increases in 2019 were 55.34%, 36.61%, 44.00%, and 30.17%, respectively, while in 2020 they were 82.30%, 54.76%, 30.19%, and 15.61%, respectively. Nitrogen content increased in all organs of the nitrogen-inefficient variety XY 508, exceeding that of the nitrogen-efficient variety ZH 311. At the N4 level, it significantly affected nitrogen content across all organs, indicating that nitrogen-inefficient varieties were more dependent on nitrogen fertilizer. Additional nitrogen is required to maintain nitrogen levels in all organs; however, the nitrogen content at maturity remains lower than that in nitrogen-efficient varieties.

**Figure 3:**
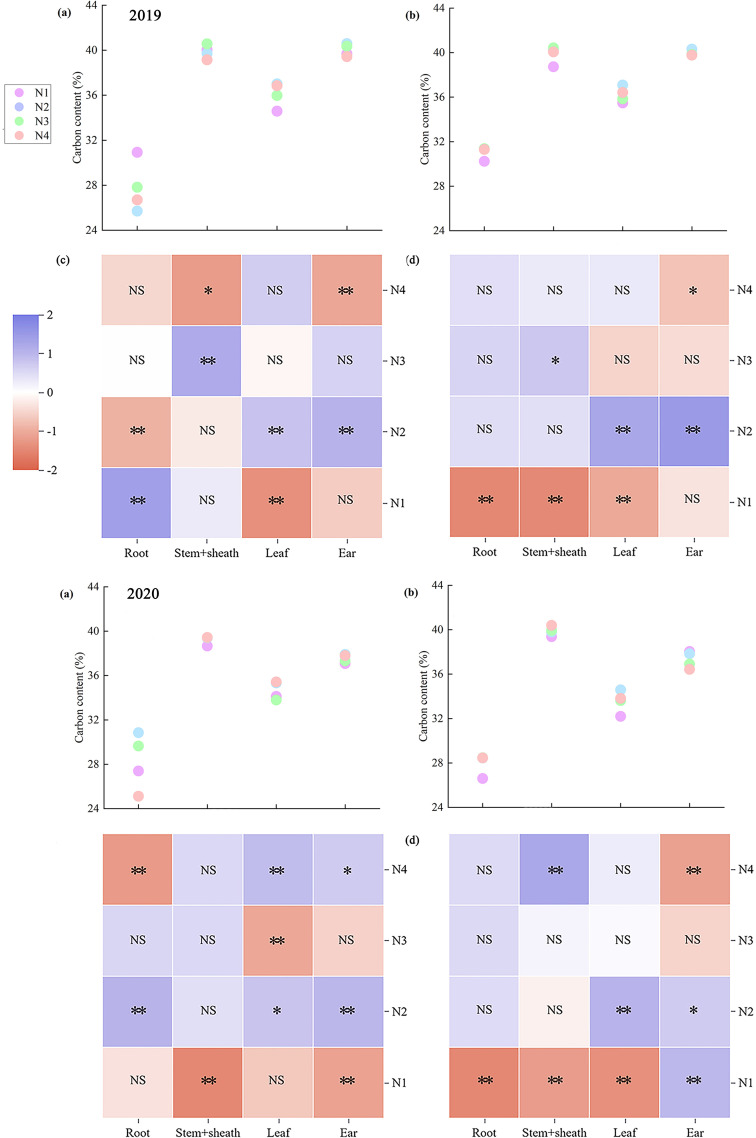
Carbon content in different organs of maize under different treatments.

Significant variations in carbon content were observed across the maize organs at maturity. Across both years, the root systems exhibited the greatest variation in carbon content, whereas the stem sheaths showed the smallest variation. Stem sheaths consistently had the highest carbon content, whereas root systems had the lowest (**Figure 3**). On average, ZH 311 exhibited higher carbon content than XY 508 in the roots, stem sheaths, leaves, and ears at maturity across the nitrogen treatments. In 2019, these differences were 10.51%, 0.04%, 0.27%, and 0.17%, respectively, while in 2020 they were higher by 0.93%, 1.63%, 3.32%, and 0.58%, respectively. Differences in carbon content among the organs were minor between the two varieties, but interannual variations were substantial and markedly distinct. Nitrogen application did not significantly alter the carbon content of mature maize organs (except for the roots in ZH311), although pronounced varietal differences persisted. Compared to the N1 treatment, the average nitrogen application treatments increased carbon content in ZH 311 roots, stem sheaths, leaves, and ears by −13.50%, −0.55%, 5.86%, and 1.11%, respectively, in 2019, and by 4.19%, 1.97%, 2.14%, and 1.58%, respectively, in 2020. For XY 508, increases in 2019 were 3.62%, 3.86%, 2.74%, and 1.58%, respectively, while in 2020 they were 6.89%, 1.72%, 5.64%, and −2.57%, respectively. XY 508 exhibited a greater increase in root carbon content than ZH 311, with smaller variations in carbon content, indicating that additional nitrogen fertilizer application favors increased root carbon content in nitrogen-inefficient varieties.

### 3.3 Differences in carbon and nitrogen accumulation among organs in maize varieties with varied nitrogen use efficiency

**Figure 4:**
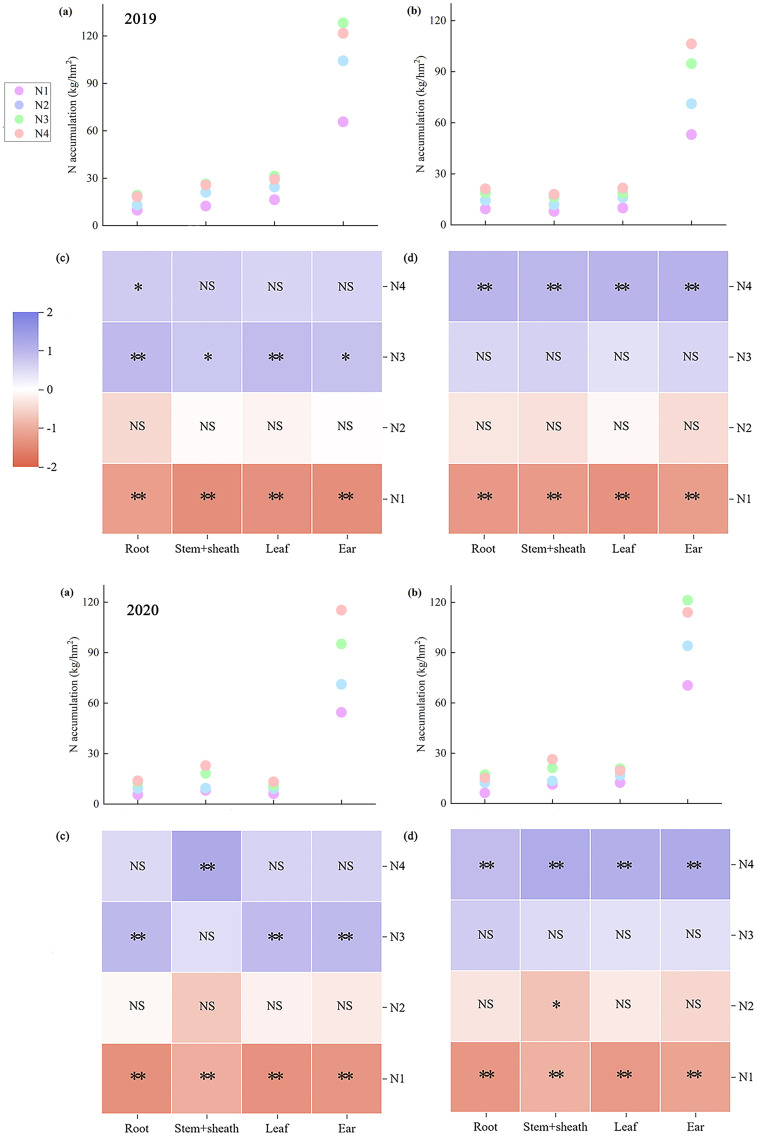
Nitrogen accumulation in different organs of maize under different treatments.

Nitrogen accumulation across maize organs at maturity exhibited marked variation. Across both years, ear nitrogen accumulation showed the greatest disparity, whereas leaf nitrogen accumulation showed the smallest difference. Moreover, ear nitrogen accumulation in ears was consistently the highest across both years, whereas root nitrogen accumulation in roots remained the lowest (**Figure 4**). On average, ZH 311 exhibited higher nitrogen accumulation in the roots (except in 2019), stem sheaths, leaves, and ears than XY 508 across nitrogen treatments. In 2019, these differences amounted to 5.40%, 57.92%, 53.12%, and 29.14%, respectively, whereas these increases were 23.00%, 23.21%, 76.04%, and 18.92%, respectively, in 2020. The nitrogen-efficient variety ZH 311 demonstrated superior nitrogen uptake capacity compared to the nitrogen-inefficient variety XY 508, with enhanced nitrogen accumulation in all organs at maturity. Nitrogen application significantly increased nitrogen accumulation in all maize organs at maturity, with pronounced varietal differences. Compared with the N1 treatment, the two-year average nitrogen accumulation in ZH 311 roots, stem sheaths, leaves, and ears increased by 97.37%, 88.83%, 64.35%, and 67.32%, respectively, whereas for XY 508, the increases were 101.98%, 101.02%, 86.33%, and 71.61%, respectively. The average increase in nitrogen accumulation across all organs in the nitrogen-inefficient variety XY 508 was higher than that in the nitrogen-efficient variety ZH 311 across both years. Furthermore, at the N4 level, differences among organs were significant, indicating that additional nitrogen fertilization favored increased nitrogen accumulation in all organs of the low-nitrogen-use-efficiency variety XY 508. However, the accumulation levels (except in 2019) remained significantly lower than those in the high-nitrogen-use-efficiency variety ZH 311.

**Figure 5:**
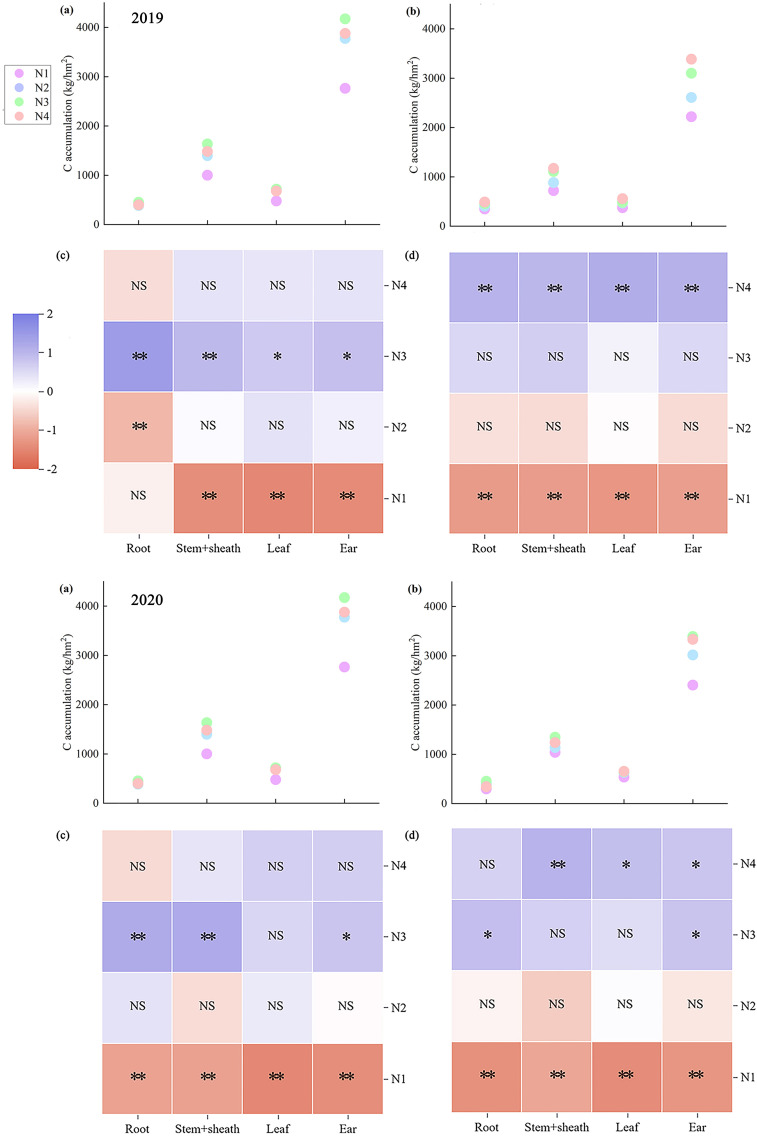
Carbon accumulation in different organs of maize under different treatments.

Significant differences in carbon accumulation were observed across the maize organs at maturity. Across both years, ear carbon accumulation exhibited the greatest variation, whereas root carbon accumulation showed the smallest variation. Moreover, ear carbon accumulation was consistently the highest, and root carbon accumulation was the lowest across both years (**Figure 5**). Across all nitrogen treatments, ZH 311 exhibited higher carbon accumulation in the roots (except in 2019), stem sheaths, leaves, and ears than XY 508 did. In 2019, these differences amounted to 2.84%, 41.99%, 36.03%, and 28.99%, respectively, and in 2020, these increases were 16.36%, 20.38%, 58.38%, and 18.35%, respectively. The nitrogen-efficient variety ZH 311 exhibited significantly higher leaf carbon accumulation than the nitrogen-inefficient variety XY 508, indicating that ZH 311 possesses a stronger photosynthetic capacity, thereby enhancing carbon accumulation in all organs at maturity. Nitrogen application significantly increased carbon accumulation in all maize organs at maturity, with pronounced varietal differences. Across both years, compared to the N1 treatment, nitrogen application increased carbon accumulation in ZH 311 roots, stem sheaths, leaves, and ears by 15.37%, 34.54%, 31.79%, and 39.06%, respectively, whereas those in XY 508 were 27.45%, 39.49%, 39.74%, and 38.55%, respectively. The nitrogen-inefficient variety XY 508 exhibited higher increases in carbon accumulation across all organs (except the ears) than the nitrogen-inefficient variety ZH 311. At the N4 level, significant differences were observed among the organs, indicating that additional nitrogen fertilization increased carbon accumulation in organs of nitrogen-inefficient varieties. However, these increases were markedly lower than those observed in nitrogen-efficient varieties.

### 3.4 Differences in carbon and nitrogen allocation among organs in maize varieties with varied nitrogen use efficiency

**Figure 6:**
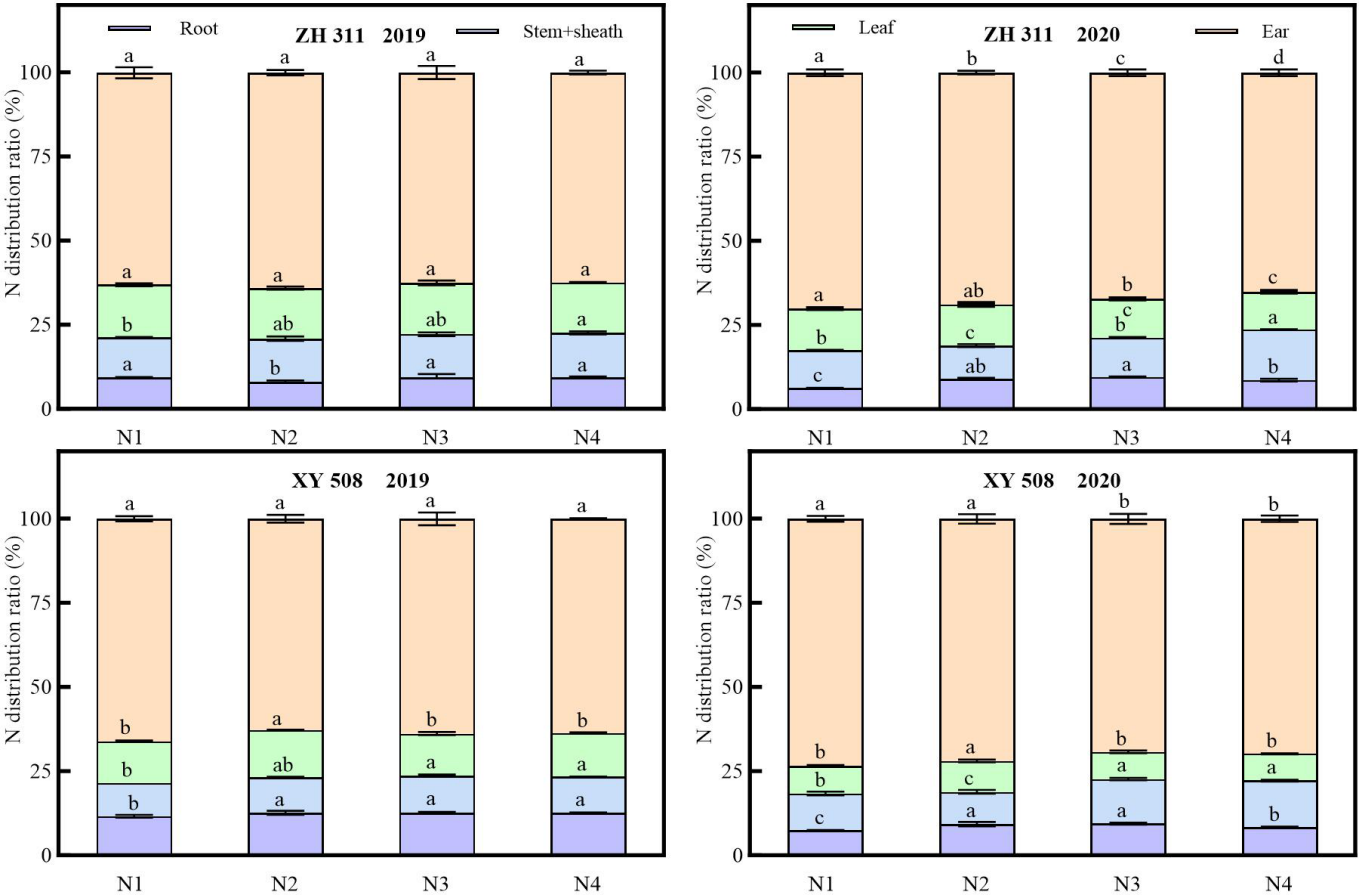
Nitrogen distribution ratio in different organs of maize under different treatments. Values followed by different lowercase letters within the same group are significantly different at the p < 0.05 level, while values with the same lowercase letter are not significantly different.

The proportion of nitrogen allocated to different organs varied significantly during maize maturity. Across both years, the ears had the highest proportion, whereas the root system had the lowest proportion (except for XY 508 in 2020; **Figure 6**). Across all nitrogen treatments, ZH 311 exhibited higher nitrogen allocation ratios in roots, stem sheaths, leaves, and ears compared to XY 508 at maturity in 2019, differing by −3.39%, 2.17%, 2.25%, and −1.04%, respectively, while in 2020, the differences were −0.25%, 0.08%, 3.46%, and −3.28%, respectively. ZH 311 exhibited higher nitrogen allocation ratios in stem sheaths and leaves than XY 508 across both years, indicating that nitrogen-efficient varieties allocate more nitrogen to stem sheaths and leaves than nitrogen-inefficient varieties, thereby facilitating efficient nitrogen flow from the source to the sink. Nitrogen application had a minor effect on the nitrogen allocation ratios at maize maturity, although significant organ-specific differences persisted. Compared to the N1 treatment, ZH 311 exhibited increased nitrogen allocation ratios in roots, stem sheaths, leaves, and ears by −0.45%, 1.14%, −0.64%, and 0.01%, respectively, in 2019, and by 2.78%, 0.93%, −0.70%, and −3.00%, respectively. For XY 508, the increases in 2019 were 1.02%, 0.82%, 0.88%, and −2.76%, respectively, whereas in 2020, they were 1.54%, 1.35%, 0.13%, and 3.05%, respectively. Increased nitrogen fertilization elevated the proportion of nitrogen allocated to maize stalk sheaths at maturity, while reducing the proportion allocated to the ears. However, the proportions allocated to leaves and roots exhibited significant interannual variability.

**Figure 7:**
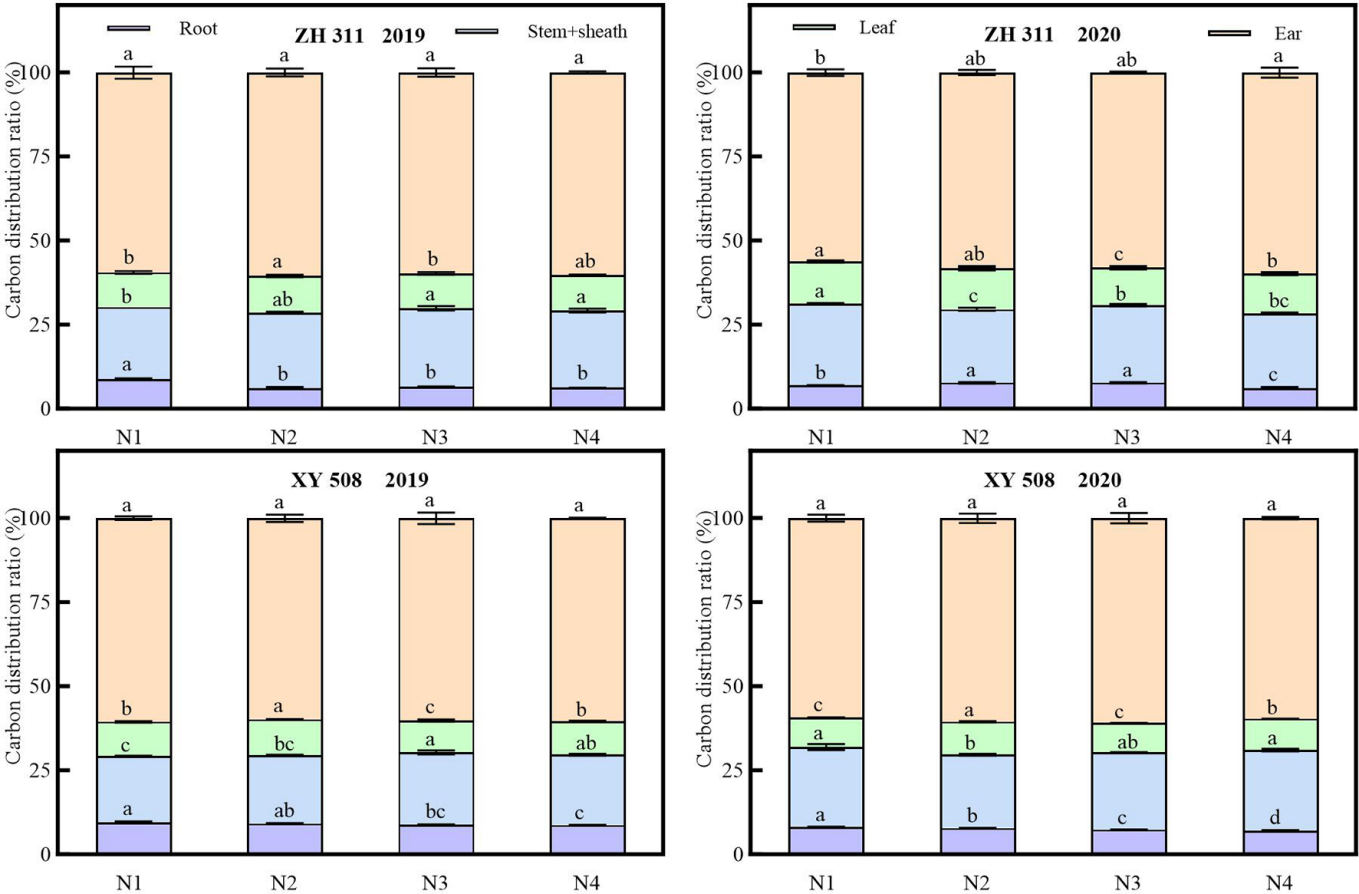
Carbon distribution ratio in different organs of maize under different treatments. Values followed by different lowercase letters within the same group are significantly different at the p < 0.05 level, while values with the same lowercase letter are not significantly different.

Significant variations in carbon allocation ratios among maize organs at maturity were observed, with the ears exhibiting the highest allocation ratio and the roots the lowest across both years (**Figure 7**). On average, across all nitrogen treatments, ZH 311 exhibited higher carbon allocation ratios than XY 508 in the roots, stem sheaths, leaves, and ears at maturity. In 2019, these differences were 2.17%, 1.99%, 0.46%, and 0.28%, respectively, while in 2020, they were 0.44%, 0.29%, 2.76%, and −2.02%, respectively. ZH 311 exhibited higher leaf carbon allocation than XY 508 across both years, whereas its root and ear carbon allocations were lower than those of XY 508, indicating differential regulation of the source–sink–flow system among varieties. Nitrogen application had no significant effect on carbon allocation among maize organs at maturity, although interannual variations were substantial. Compared to the N1 treatment, ZH 311 exhibited increased nitrogen allocation ratios in roots, culms, leaves, and ears by −2.43%, 1.43%, 0.29%, and 0.77%, respectively, in 2019, and by 0.26%, −1.91%, −0.85%, and 2.50%, respectively; for XY 508, the increases were −0.60%, 1.19%, −0.15%, and −0.46%, respectively, in 2019, and −0.70%, −0.90%, 0.51%, and 1.08%, respectively, in 2020.

### 3.5 Differences in carbon-to-nitrogen ratios among organs in maize varieties with varied nitrogen use efficiency

**Figure 8:**
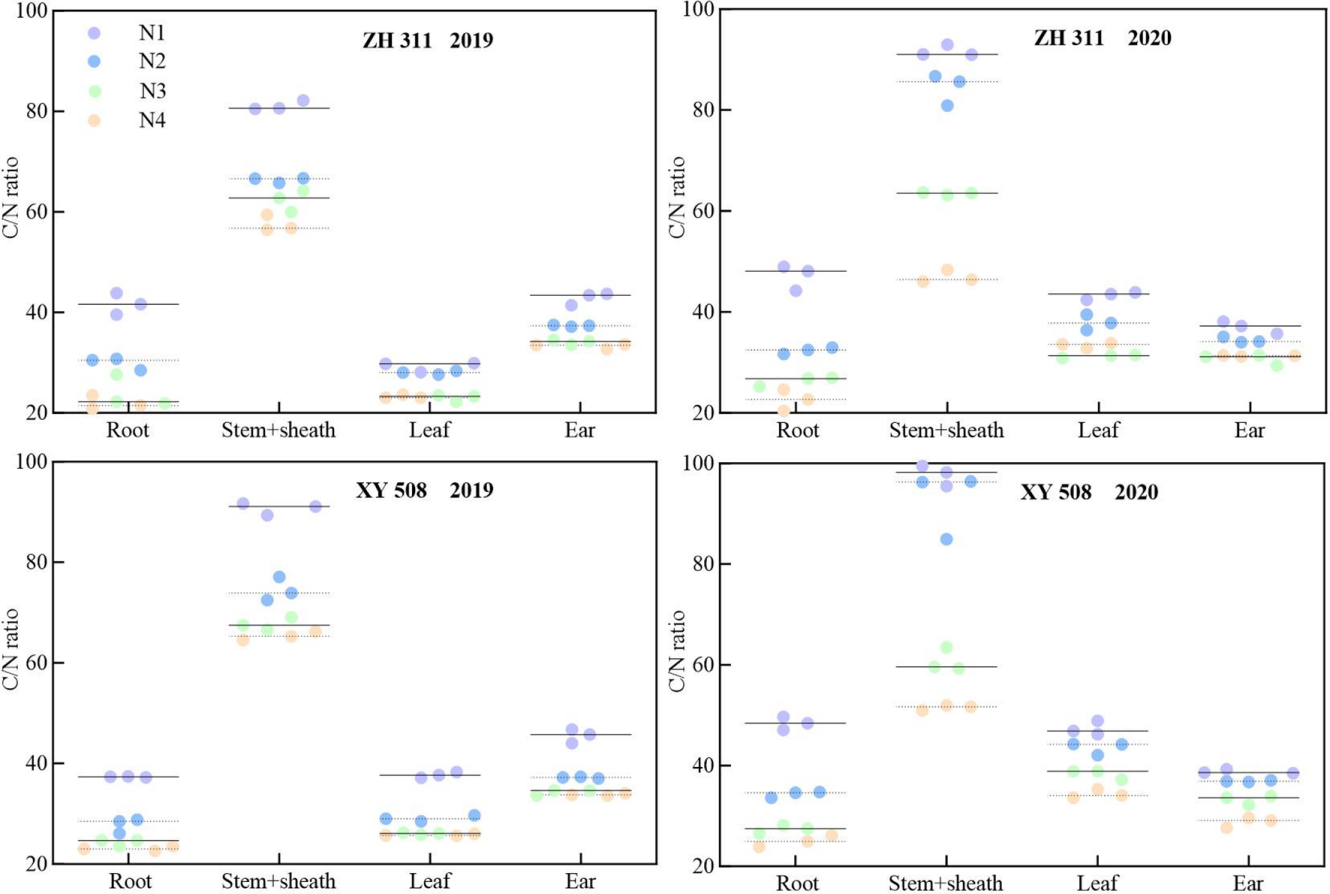
C/N ratio in different organs of maize under different treatments.

Significant differences in the carbon-to-nitrogen ratios were observed across maize organs at maturity. Across both years, the stem sheath exhibited the highest carbon-to-nitrogen ratio, with considerable variation, whereas the root system displayed the lowest ratio (**Figure 8**). On average, across all nitrogen treatments, ZH 311 exhibited lower carbon-to-nitrogen ratios than XY 508 in the roots (except in 2019), stems, leaves, and ears. In 2019, these ratios were lower by 1.22%, 7.76%, 3.80%, and 0.83%, respectively, while in 2020 they were lower by 1.68%, 4.05%, 4.40%, and 1.10%, respectively. This indicates that ZH 311 possesses a stronger carbon-nitrogen balance capacity than XY 508, which maintains carbon-nitrogen ratios across the organs. The carbon–nitrogen ratios in all organs of nitrogen-treated maize significantly decreased at maturity, with pronounced varietal differences. Compared to the N1 treatment, ZH 311 exhibited average reductions in carbon-to-nitrogen ratios of 16.41%, 19.02%, 4.51%, and 7.94% for roots, stem sheaths, leaves, and ears, respectively, in 2019, while in 2020 these reductions were 19.98%, 26.71%, 9.08% and 4.87%, respectively. For XY 508, the reductions in 2019 were 12.26%, 21.53%, 10.74%, and 10.42%, respectively, and in 2020, 19.49%, 29.44%, 8.62%, and 5.80%, respectively. Over the two years, both varieties exhibited the greatest carbon-to-nitrogen ratio reduction in the stems and sheaths, with the smallest reduction in the ears. Moreover, the nitrogen-efficient variety ZH 311 showed smaller reductions in both stems and ears than XY 508. This indicates that during nitrogen application, the nitrogen-efficient variety ZH 311 better maintains the ‘flow’ of nutrients.

### 3.6 Differences in yield and yield components among maize varieties with varied nitrogen use efficiency

**Figure 9:**
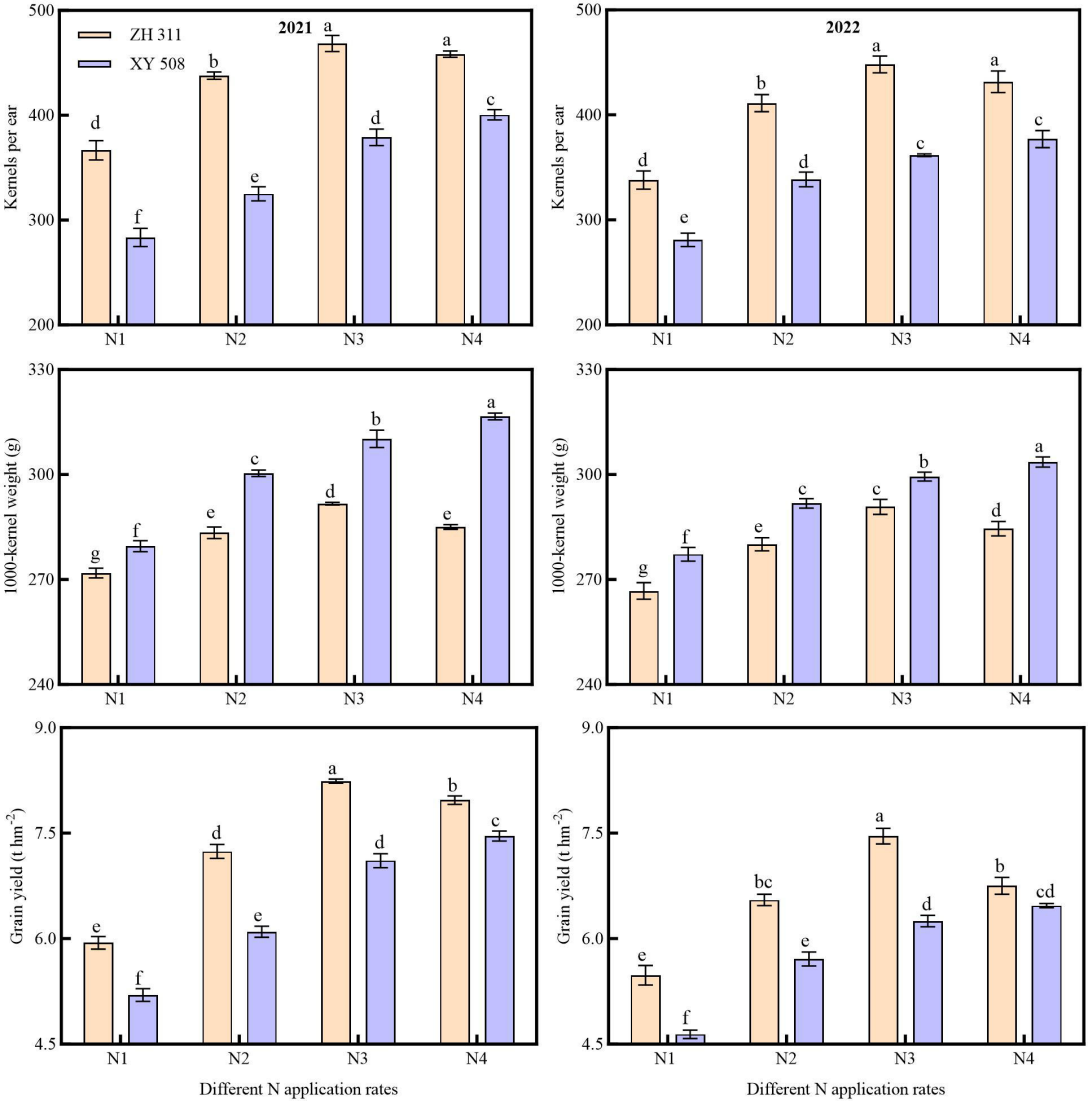
Maize grain yield and its composition under different treatments. Values followed by different lowercase letters within the same group are significantly different at the p < 0.05 level, while values with the same lowercase letter are not significantly different.

The two maize varieties exhibited significant differences in ear grain number, thousand-grain weight, and yield. Across both years, ZH 311 demonstrated higher ear grain numbers and yields than XY 508, though its thousand-grain weight was lower than that of XY 508 (**Figure 9**). Across all nitrogen treatments, ZH 311 exhibited higher ear grain numbers, yield, and thousand-grain weight by 24.73%, 13.66%, and −6.20%, respectively, in 2019, and by 19.92%, 13.78%, and −4.26%, respectively, in 2020. Nitrogen application significantly increased maize ear grain number, thousand-grain weight, and yield, with pronounced varietal differences. Compared to the N1 treatment, the average across all nitrogen treatments showed that ZH 311 increased the ear grain number, thousand-grain weight, and yield by 24.06%, 5.45%, and 31.53%, respectively, in 2019, and by 27.32%, 6.89%, and 26.36%, respectively, in 2020. XY 508 exhibited increases of 29.89%, 10.56%, and 32.35% in 2019, and 27.75%, 7.59%, and 32.53% in 2020, respectively. The rate of increase in panicle grain number, thousand-grain weight, and yield for XY 508 exceeded those for ZH 311, indicating superior nitrogen use efficiency. Specifically, under higher nitrogen application rates, XY 508 demonstrated a greater capacity to convert nitrogen into biomass and yield, resulting in more pronounced gains in panicle grain number, thousand-grain weight, and yield.

**Figure 10:**
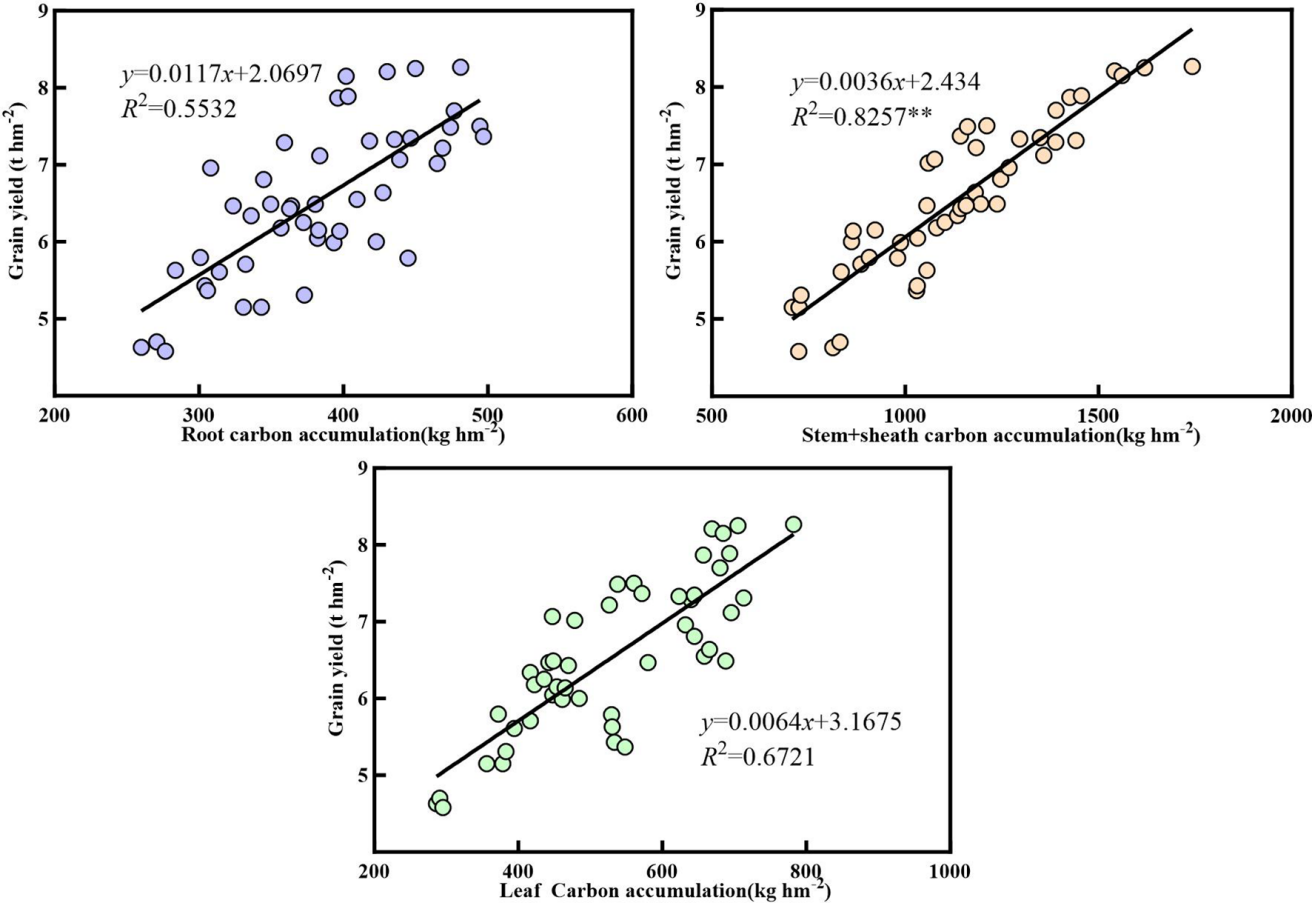
Correlation between nitrogen accumulation in vegetative organs and grain yield.

**Figure 11:**
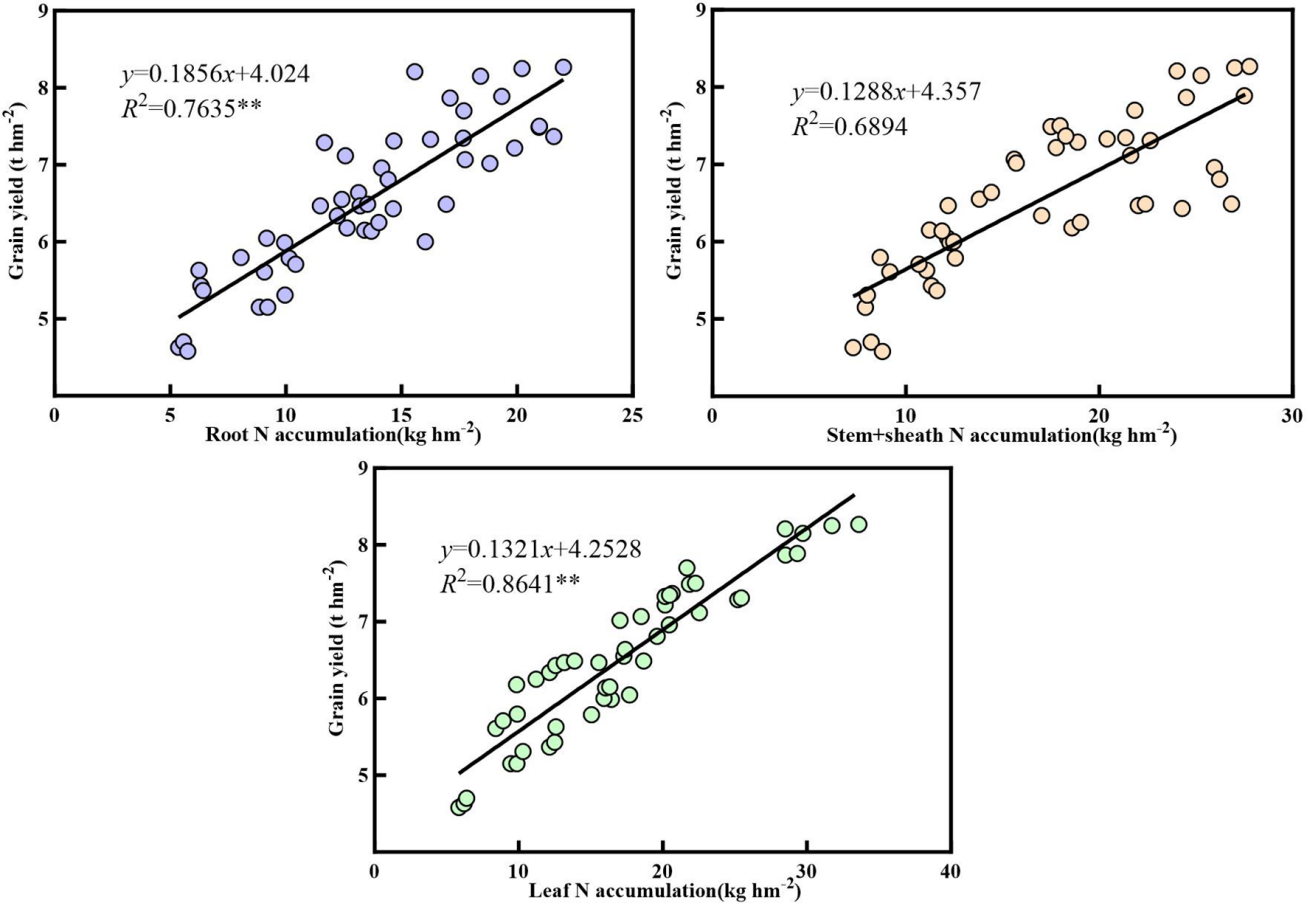
Correlation between carbon accumulation in vegetative organs and grain yield.

**Figure 12:**
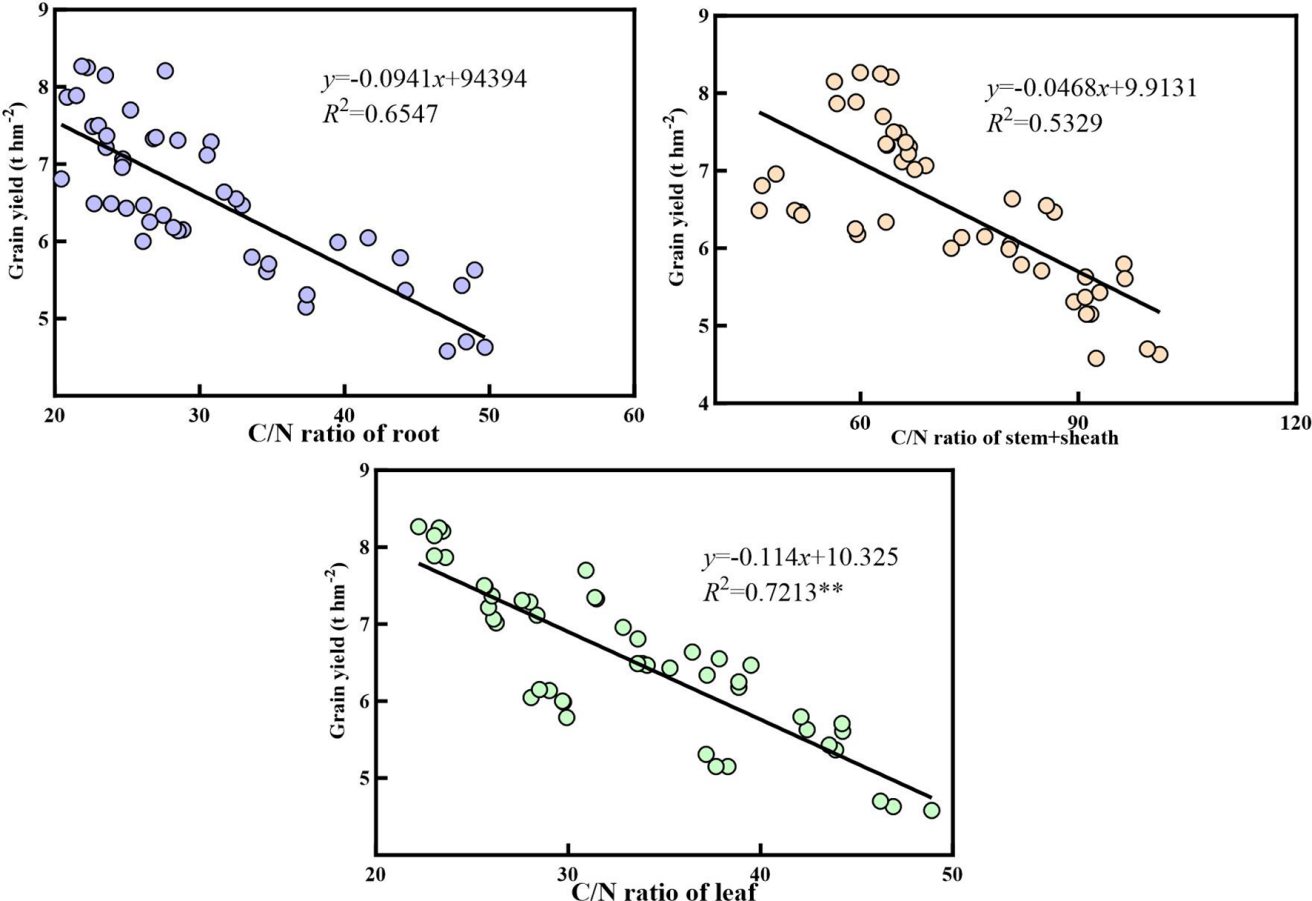
Correlation between C/N ratio in vegetative organs and grain yield.

**Figures 10**–**12** demonstrate that nitrogen accumulation, carbon accumulation, and the carbon-to-nitrogen ratio in the nutritional organs of maize at maturity significantly influenced grain yield. Nitrogen accumulation in the roots, stem sheaths, and leaves during maize maturity was positively correlated with grain yield. Leaf nitrogen accumulation exhibited the strongest correlation with grain yield (*R*² = 0.8641**), whereas stem sheath nitrogen accumulation showed the weakest correlation (*R*² = 0.6894). Carbon accumulation in the roots, stem sheaths, and leaves during maize maturity was positively correlated with grain yield. Carbon accumulation in stem sheaths exhibited the strongest correlation with grain yield (*R*² = 0.8257**), whereas carbon accumulation in the roots showed the weakest correlation (*R*² = 0.5532). At maize maturity, the carbon-to-nitrogen ratios in the roots, sheaths, and leaves were positively correlated with grain yield. Among these, the leaf carbon-to-nitrogen ratio exhibited the strongest correlation with grain yield (*R*² = 0.7213**), whereas the sheath carbon-to-nitrogen ratio showed the weakest correlation (*R*² = 0.5239).

## 4 Discussion

### 4.1 Differences in carbon and nitrogen nutrition characteristics among organs of maize varieties with varied nitrogen use efficiency

Nitrogen participates in the synthesis of plant amino acids, proteins, and nucleic acids (Wang, 2023a), whereas carbon comprises the organic matter (such as carbohydrates, fats, and proteins) and structural components (such as cell walls, leaves, and root systems) of plants (Zhang et al., 2025). Consequently, carbon and nitrogen nutrition are crucial for crop yield. Plants with higher nitrogen content exhibit greater resource competitiveness (Wang et al., 2023b), whereas elevated carbon content indicates superior photosynthetic efficiency, enabling increased carbohydrate synthesis (Tan et al., 2024). The present study revealed that ZH 311 exhibited higher nitrogen content in the stem sheaths, leaves, and spikes than XY 508 at maturity. However, the root nitrogen content was lower than that of XY 508. The greatest difference in nitrogen content was observed between the leaves of the two varieties, indicating that nitrogen-efficient varieties optimize nitrogen allocation among organs during the later growth stages compared to nitrogen-inefficient varieties. This allows for better maintenance of the aboveground nitrogen content, delays the senescence of vegetative organs (particularly leaves), and sustains higher photosynthetic productivity, consistent with previous studies (Li, 2017; Xie, 2018; Niu, 2021). At maturity, differences in carbon content among the organs were not pronounced between the two varieties, but substantial interannual variation was observed, consistent with the findings of Niu et al (Song, 2023). Interannual variations in meteorological factors (temperature, CO_2_ concentration, precipitation, and light intensity) influence the photosynthetic and carbon metabolic processes, leading to greater interannual differences in organ carbon content. Varietal differences in carbon metabolism capacity primarily manifest as differences in carbon accumulation (biomass) rather than in carbon content. Nitrogen application substantially increased the nitrogen content in all maize organs at maturity, whereas the carbon content showed smaller variations. Consequently, the carbon-to-nitrogen ratio decreased with increasing nitrogen application, consistent with the findings of Lv et al (Lv et al., 2008). for summer maize. XY 508 exhibited greater increases in nitrogen content across all organs than ZH 311, indicating that nitrogen application more effectively improved the nitrogen nutrition characteristics in the organs of nitrogen-inefficient varieties. This aligns with the finding of Cao et al (Cao et al., 2012). that nitrogen-inefficient rapeseed varieties showed more pronounced improvements in nitrogen nutrition characteristics than nitrogen-efficient varieties following nitrogen application. Moreover, after nitrogen application, XY 508 exhibited a markedly greater increase in root carbon content than ZH 311 did. This indicated that nitrogen-inefficient varieties require higher nitrogen nutrition to sustain root growth and ensure root carbon storage, consistent with the findings of Fan et al (Fan, 2008).

The carbon-to-nitrogen ratio (C/N) not only reflects nutrient utilization strategies in response to environmental changes but also indicates plant growth status. An excessively high C/N ratio signifies insufficient nitrogen supply and growth inhibition, whereas an excessively low ratio leads to imbalanced growth, affecting the transition from vegetative to reproductive growth (Chen et al., 2023a). This study found significant differences in C/N ratios among maize organs at maturity, with the highest C/N ratio in stem sheaths and the lowest in the roots. This is consistent with the findings of Zhang et al. (Zhang and Cui, 2020a) As a ‘carbon reservoir,’ the stem sheath serves as the primary storage site for photosynthetic products, whereas the root system, functioning as a ‘nitrogen hub,’ efficiently absorbs and metabolizes nitrogen. Consequently, the stem sheath exhibited a substantially higher C/N ratio than the roots. The nitrogen-efficient variety ZH 311 exhibited lower C/N ratios in the roots, sheaths, leaves, and ears than the nitrogen-inefficient variety XY 508, consistent with the findings of Lv et al (Lv et al., 2008). Nitrogen-efficient varieties exhibit superior coordination of carbon-nitrogen metabolism, along with enhanced nitrogen utilization and transport capacity. This enables them to maintain nitrogen content in vegetative organs while ensuring relatively stable carbon transport, resulting in lower carbon-to-nitrogen ratios (C/N). This further demonstrates that nitrogen-efficient varieties sustain nitrogen nutrition in vegetative organs more effectively than nitrogen-inefficient varieties during the later growth stages. At maturity, the C/N ratios of all organs decreased in nitrogen-treated maize. For both varieties across the two years, the greatest reduction occurred in the stem sheath, whereas the smallest reduction was observed in the ear. This indicates that the stem sheath, acting as a ‘buffer reservoir,’ rapidly releases carbon and nitrogen to sustain ear development. In contrast, the ear, functioning as the ‘terminal reservoir,’ maintains carbon-nitrogen balance through metabolic prioritization. This is consistent with the findings of Chen et al (Chen et al., 2023b). At maturity, the nitrogen-efficient variety ZH 311 exhibited smaller decreases in both stem sheath and ear carbon-to-nitrogen ratios than the nitrogen-inefficient variety XY 508. This indicates superior ‘flow’ capacity in nitrogen-efficient varieties relative to nitrogen-inefficient ones during maturity, enabling better maintenance of plant carbon-nitrogen balance. This finding is consistent with that reported by Mi et al (Mi et al., 2007). Correlation analysis revealed that the carbon-to-nitrogen ratios of the maize nutritional organs (roots, stem sheaths, and leaves) were negatively correlated with yield, with leaves showing a significant correlation (0.7213**). This is consistent with the findings of Zhang et al. (Zhang et al., 2013b). Nitrogen-efficient varieties maintain lower C/N ratios in the roots, stem sheaths, and leaves than nitrogen-inefficient varieties, constituting a key mechanism for their yield enhancement.

### 4.2 Differences in carbon and nitrogen accumulation and allocation among organs in maize varieties with different nitrogen use efficiency

The crop yield depends on the accumulation and distribution of photosynthetic assimilates across different organs (Wei et al., 2017). Previous studies on crops such as wheat (Wang et al., 2023c), sorghum (Ma et al., 2024), and bitter buckwheat (Xia et al., 2020) have demonstrated that nitrogen application enhances nitrogen translocation rates in vegetative organs, increases carbon and nitrogen accumulation, and consequently significantly boosts yield. Moreover, the yield was significantly positively correlated with carbon and nitrogen accumulation. The results of this study indicate that during maize maturity, carbon and nitrogen accumulation were highest in the ears and lowest in the roots, consistent with the findings of Zhang et al. (2013a) regarding the distribution patterns of carbon and nitrogen accumulation in summer maize at maturity (highest in ears and lowest in roots). Further analysis revealed that nitrogen accumulation in the roots, stem sheaths, and leaves during maize maturity was positively correlated with grain yield. This correlation was significant for roots (0.7635**) and leaves (0.8641**), consistent with the findings of Ma (Ma, 2017). Nitrogen accumulation in the roots improves root structure and enhances nitrogen uptake capacity, thereby increasing yield (Li and Guo, 2023b). Leaf nitrogen accumulation increases biomass through photosynthesis, thereby increasing yield (Sui et al., 2018). Carbon accumulation in the roots, stem sheaths, and leaves during maize maturity was positively correlated with grain yield, with stem sheaths (0.8257**) reaching significant levels. This aligns with the findings of Fan et al. (2010) in wheat. The stem sheath functions as a crucial ‘carbon reservoir’ and ‘nutrient transport hub’ during the late growth stage, where enhanced NSC transport to grains contributes to increased yield. Furthermore, ZH 311 exhibited higher carbon and nitrogen accumulation in the roots, stem sheaths, and leaves than did XY 508 across both years, consistent with the findings of Chang et al. (2021) for low-nitrogen-efficient maize (PH6WC) and low-nitrogen-inefficient maize (ZY118). Nitrogen-efficient varieties maintain carbon and nitrogen nutrition in vegetative organs, optimize root structure, enhance nitrogen accumulation, coordinate photosynthesis-carbon metabolism, and increase carbon accumulation better than nitrogen-inefficient varieties. Nitrogen application significantly increased carbon and nitrogen accumulation in the roots, stem sheaths, and leaves of all maize varieties, although XY 508 exhibited greater increases than ZH 311. This aligns with the findings of Zhang, (2020) in summer maize, indicating that increased nitrogen fertilization is more beneficial for enhancing carbon and nitrogen accumulation in nitrogen-inefficient varieties. These varieties require higher nitrogen application rates to maintain carbon and nitrogen accumulation; however, their accumulation levels remain significantly lower than those of nitrogen-efficient varieties under identical nitrogen conditions.

Different crop organs exhibit distinct demands for carbon and nitrogen nutrition. Rational allocation enhances utilization efficiency and consequently boosts yield (Wang, 2018). Hu et al. (2021) observed significant differences in carbon-nitrogen allocation during maize maturity, with substantially reduced proportions in stems and leaves, whereas ears were enlarged to accommodate the increased yield. The present study indicates that during maize maturity, the carbon-to-nitrogen allocation ratio is the highest in the ear and lowest in the root system, consistent with Guo’s (2023) findings on carbon-nitrogen allocation regulation in winter wheat. A high allocation ratio to the ear reduces nutrient wastage, enhances the transport efficiency of photosynthetic products, balances the carbon-to-nitrogen ratio, and improves grain yield and quality. During the ZH 311 maturity stage, the leaf carbon-to-nitrogen allocation ratios consistently exceeded those of XY 508, consistent with the findings of Li et al. (2018). Elevated leaf carbon-to-nitrogen ratios at maturity enhance photosynthetic and carbon assimilation efficiencies, boosting the accumulation and distribution of photosynthetic products and nutrients, thereby increasing grain yield. Further analysis revealed that nitrogen application reduced the nitrogen allocation ratio in XY 508 ears while increasing the carbon allocation ratio in ZH 311 ears. However, the carbon-to-nitrogen ratios in other vegetative organs showed no significant changes, consistent with the findings of Ding et al. (2024) in maize. As a ‘storage organ’ in maize, carbon and nitrogen accumulation in the ear directly determines grain yield. Following nitrogen application, nitrogen-efficient varieties can effectively enhance leaf photosynthetic rates and increase the ‘storage capacity’ of the ear, thus directing newly generated carbon sources to the ear to increase grain yield.

## 5 Conclusion

At maturity, ZH 311 exhibited higher nitrogen content in stem sheaths, leaves, and ears than XY 508, whereas its root nitrogen content was lower than that of XY 508. However, no significant differences were observed in the carbon content among the roots, stem sheaths, leaves, and ears of the two varieties at maturity, resulting in lower C/N ratios in all ZH 311 nutritional organs than in XY 508. Nitrogen-efficient maize cultivar ZH 311 optimizes nitrogen allocation to photosynthetic and yield-forming organs, maintaining lower vegetative C/N ratios and stable carbon-nitrogen accumulation under moderate nitrogen supply, whereas inefficient XY 508 relies on high nitrogen inputs to meet metabolic demands; leaf nitrogen and stem sheath carbon accumulation are key yield determinants. Further analysis revealed that the C/N ratios of the maize roots, stem sheaths, and leaves were negatively correlated with grain yield. This indicates that maintaining a low C/N ratio in vegetative organs is a key physiological mechanism that enables nitrogen-efficient varieties to yield more than the nitrogen-inefficient varieties. Increased nitrogen fertilization significantly elevated carbon and nitrogen accumulation in maize roots, sheaths, and leaves. However, XY 508 exhibited greater increases in carbon and nitrogen accumulation across all organs than ZH 311, indicating that nitrogen fertilization more strongly promotes carbon and nitrogen accumulation in the nutritional organs of nitrogen-inefficient varieties. Moreover, XY 508 requires higher nitrogen application rates to sustain its carbon-nitrogen accumulation demands. At identical nitrogen levels, the nitrogen-efficient variety ZH 311 exhibited substantially higher carbon-nitrogen accumulation in the roots, stem sheaths, and leaves than XY 508, demonstrating a pronounced carbon-nitrogen accumulation advantage over nitrogen-inefficient varieties. Correlation analysis revealed that carbon and nitrogen accumulation in the roots, stem sheaths, and leaves at maize maturity positively correlated with grain yield. Among these, leaf nitrogen accumulation (*R*² = 0.8641**) and stem sheath carbon accumulation (*R*² = 0.8257**) had the strongest influence on grain yield.

## Data Availability

The original contributions presented in the study are included in the article/supplementary material. Further inquiries can be directed to the corresponding authors.
